# The Transformation of Hg^2+^ during Anaerobic S^0^ Reduction by an AMD Environmental Enrichment Culture

**DOI:** 10.3390/microorganisms11010072

**Published:** 2022-12-27

**Authors:** Yuhang Zhou, Yue Liu, Hongchang Liu, Zhenyuan Nie, Yirong Wang, Lu Chen

**Affiliations:** 1 School of Minerals Processing and Bioengineering, Central South University, Changsha 410083, China; 2 Key Lab of Biometallurgy of Ministry of Education of China, Central South University, Changsha 410083, China

**Keywords:** acid mine drainage, AMD environmental enrichment culture, microbial S^0^ reduction, Hg^2+^ transformation, microbial community structure

## Abstract

Mercury (Hg) is a highly toxic and persistent heavy metal pollutant. The acid mine drainage (AMD) environment in sulfide-mining areas is a typical Hg pollution source. In this paper, the transformation of Hg^2+^ during anaerobic S^0^ reduction by an AMD environmental enrichment culture was studied by multiple spectroscopic and microscopic techniques. The experimental results showed that the microbial S^0^ reduction of the AMD enrichment culture was significantly inhibited in the presence of Hg^2+^. The results of cell surface morphology and composition analysis showed that there was obvious aggregation of flocculent particles on the cell surface in the presence of Hg^2+^, and the components of extracellular polymeric substances on the cell surface changed significantly. The results of surface morphology and C/S/Hg speciation transformation analyses of the solid particulate showed that Hg^2+^ gradually transformed to mercuric sulfide and Hg^0^ under anaerobic S^0^ reduction by the AMD enrichment culture. The microbial community structure results showed that Hg^2+^ significantly changed the enrichment community structure by decreasing their evenness. The dominant microorganisms with S^0^ reduction functions are closely related to mercury transformation and are the key driving force for the transformation of substrate solid particulate and cellular substances, as well as the fixation of Hg^2+^.

## 1. Introduction

Heavy metal pollution has become among the most serious environmental problems in acid mine drainage (AMD) along with the mining of metal sulfide ores, causing potential threats to human health [1]. Among the numerous heavy metal elements existing in AMD, mercury (Hg) is among the most toxic heavy metal pollutants, with global distribution and persistent pollution. Hg can occur in various states and species in the environment, including the oxidation states of Hg(0) (elemental mercury), Hg(I) (mercurous), and Hg(II) (mercuric) [2], and the organic forms of methyl mercury (MeHg) and ethyl mercury (EtHg), which determine the toxicity and environmental impact of Hg [3]. The transformation of Hg species in a polluted environment has become a research hotspot in recent decades [4,5,6].

Microorganisms play an important role in the transformation of Hg species, mainly including the oxidation, reduction, methylation, and demethylation of Hg [7,8]. Under anaerobic conditions, microorganisms can tolerate organic and inorganic mercury in the environment by reducing and methylating Hg^2+^ and demethylating MeHg [9,10]. Microbial resistance to MeHg transported into cells relies on proton-assisted cleavage of the Hg-C bond of MeHg by organomercury lyase (MerB) and the reduction of mercuric Hg^2+^ into nontoxic Hg^0^ by mercuric reductase (MerA) in the *mer* operon, and inorganic mercury and organic mercury finally diffuse to the outside of cells in the form of Hg^0^ and are released into the atmosphere [11,12,13]. For example, *Acidithiobacillus ferrooxidans*, which is abundant in the AMD environment, can convert toxic Hg^2+^ into relatively inert Hg^0^ by MerA, which is necessary for this bacterium to survive in the Hg-polluted AMD environment [14].

AMD is a typical extreme acidic environment rich in Hg^2+^, in which the microbial community structure is different from that of other environments. Niane et al. studied mercury-resistant bacteria in contaminated aquatic sediments in the Kedougou region, Senegal, and found that the microbial community structure characteristics of the AMD environment tend to be tolerant to high Hg concentrations compared with other freshwater areas [15]. Of note, microorganisms in the AMD environment are closely related to the iron-sulfur cycle. Among them, microbially mediated sulfur reduction is of great significance in the remediation of heavy metal pollution. For example, sulfur-reducing bacteria are widely used in the treatment of heavy metals in landfill sites, which is primarily due to their ability to effectively immobilize Hg^2+^ and affect the bioavailability of Hg^2+^ by forming HgS [16,17]. The main sulfur species entering the AMD environment in sulfide-mining areas are sulfate and S^0^, formed by the chemical and/or microbial oxidation of pyrite. These sulfur species are easily reduced and oxidized under the action of sulfur-reducing and sulfur-oxidizing microorganisms. Microbial sulfur reduction usually occurs under anaerobic conditions with sulfate or S^0^ as the electron acceptor and generates low-valence sulfur such as S^2−^, which can further react with heavy metal ions to form metal sulfide precipitates, thus reducing the toxicity of heavy metal ions and inhibiting their mobility [18,19]. Compared with microbially mediated sulfate reduction, there are relatively few studies on microbially mediated S^0^ reduction for AMD treatment [20,21], and the effects of Hg^2+^ on S^0^ reduction mediated by acidophiles and the relevant mechanisms are worthy of study.

Therefore, this work focused on mercury transformation during anaerobic S^0^ reduction by an AMD environmental enrichment culture. On the basis of the effect of Hg^2+^ on the anaerobic S^0^ reduction of the AMD environment enrichment culture, the microbial diversity of the enrichment culture in the process was analyzed. The mechanisms of microbially mediated anaerobic S^0^ reduction related to mercury transformation were further studied by multiple spectroscopy and microanalysis techniques. The relevant results not only provide a reference for screening mercury-tolerant microorganisms but also provide basic information for mercury pollution control and remediation in heavy metal-polluted areas.

## 2. Materials and Methods

### 2.1. Preparation of the AMD Enrichment

The sediment sample was collected from the Dabaoshan mine site in Guangdong Province, China, and an approximately 120 g sediment sample was added to a homemade bioreactor (Figure S1) containing 12 L 9K basal medium with 1 g/L S^0^ for incubation at ambient temperature. The 9K basic medium consisted of (NH_4_)_2_SO_4_ 3.0 g/L, KCL 0.1 g/L, K_2_HPO_4_ 0.5 g/L, MgSO_4_·7H_2_O 0.5 g/L, Ca(NO_3_)_2_ 0.01 g/L, and CH_3_COONa 20 mM, and the initial pH was adjusted to 2.8 with 2 M H_2_SO_4_ solution. After 60 days of incubation, the bacteria at the bottom of the bioreactor were collected through the sampling port and enriched in an anaerobic incubator (LAI-3T-N1) at 30 °C, with solely the addition of S^0^ as the energy substrate. After 3–4 generations of cultivation, an AMD enrichment culture with high S^0^ reduction activity was obtained by monitoring the dissolved S^2−^ concentrations during cultivation.

### 2.2. Experimental Setup

The AMD environmental enrichment culture was cultured in 250 mL conical flasks containing 100 mL sterile 9K basic medium with an initial pH of 2.8 and an initial cell density of 8 × 10^7^ cells/mL. The concentration of Hg^2+^ used in the present study was 2 mg/L, which was obtained by a pre-experiment investigating the effect of different concentrations of Hg^2+^-[Hg(NO_3_)_2_] on microbial aerobic growth (Figure S2). The experimental groups were statically cultured in an anaerobic incubator at 30 °C without Hg^2+^ and with 2 mg/L Hg^2+^, and each experimental group was tested in triplicate. During the experiment, the solution samples, solid particulate and cells were collected at different times for further analysis. Sampling operations were carried out in the anaerobic incubator, where the solution samples were obtained at 1–2 day intervals in the initial stage and 3–5 days in the later stage of exponential growth. The solid particulate was collected and frozen immediately in an ultralow temperature freezer (−80 °C) and then dried under vacuum conditions to analyze their morphology and composition. The cells were collected by the differential velocity centrifugation method, and then the microbial morphology and surface composition were analyzed. Each experiment was performed in triplicate, and the mean value ± standard deviation of the data determined was presented for the following analyses.

### 2.3. Analytical Methods

#### 2.3.1. Physical and Chemical Characterization of the Culture Liquor

The cell density was directly counted by light microscopy (Nexcopy NE900, Yongxin, Ningbo, China) with a blood corpuscle counter (XB-K-25, Qiujing, Shanghai, China). The concentration of dissolved ΣS^2−^ ([S^2−^]_aq_) was determined spectrophotometrically by the methylene blue method, and the concentration of dissolved Hg^2+^ ([Hg^2+^]_aq_) was measured by an inductively coupled plasma emission spectrometer.

#### 2.3.2. Surface Morphology and Chemical Composition of Solid Particulate and Microbial Cells

The solid particulate and cell samples were obtained in the experiment and freeze-dried. The surface morphology and element distribution of the solid particulate and the cells in different periods were characterized by the scanning electron microscopy (SEM) and X-ray energy dispersive spectroscopy (EDS) (MIRA 3 LMU, Tescan, Brno, Crech Republic). Briefly, the samples were prefixed with 2.5% formaldehyde for 4 h, dehydrated with graded ethanol, and introduced into the SEM chamber for observation. The internal structure and element distribution of the single cell was characterized by transmission electron microscopy (TEM)-EDS (Talos F200X, Thermo Fisher, Sunnyvale, CA, USA). Before the TEM observation, the microbial cells were prefixed with 2.5% formaldehyde for 4 h, immersed with 1% osmic acid for 2 h, dehydrated with graded ethanol, transferred through epichlorohydrin, embedded in epoxy resin, and cut into ultrathin section. The surface composition and functional groups of the solid particulate and the cells were characterized by FT-IR. The FT-IR spectra were collected in the range of 4000–500 cm^−1^ by an FT-IR spectrometer (Nexus 670, Nicolet, Madison, WI, USA) after mixing 0.9 mg of each sample with 80 mg of KBr and pressing the mixture into a pellet. The phase composition of the solid particulate was analyzed by Raman spectroscopy. Briefly, the Raman spectra were recorded at room temperature in the range of 200–4000 cm^−1^ by a Raman spectrometer (DXR, Thermo Fisher, Sunnyvale, CA, USA).

#### 2.3.3. C, S and Hg Speciation on the Surface of Solid Particulate

The C, S and Hg speciation transformation on the surface of the solid particulate was analyzed by X-ray photoelectron spectroscopy (XPS) with an X-ray photoelectron spectrometer (ESCALAB Xi+, Thermo Fisher, Sunnyvale, CA, USA). All photoelectron binding energies were referenced to C 1*s* adventitious contamination peaks set at 285.0 eV, and the linear combination of the measured spectrum was fitted with CASAXPS software (v2.3.16).

#### 2.3.4. Analysis of Microbial Diversity

Microbial diversity was determined by Illumina high-throughput sequencing at Magigene Co., Ltd., Guangzhou, China. The total DNA of the cell samples was extracted by the total DNA Extraction Kit (DNeasy Powersoil Kit, Qiagen, Hilden, Germany). The integrity and purity of DNA were detected by 1% agarose gel electrophoresis, and the concentration and purity of DNA were detected by a NanoDrop One. The 16S rRNA gene was amplified with barcode primers and PremixTaq (TaKaRa, Shiga, Japan) and sequenced on an Illumina HiSeq platform. The sequencing was completed by Guangdong Meige Gene Technology Co., Ltd., and the reads without correct barcode information and an essential quality score of Q20 or higher were removed using Mothur, and then mapped against consensus 16S RNA V3–V4 sequences, yielding 87,283 and 88,075 filtered reads on average for the groups without and with Hg^2+^, respectively, accounting 98.9 and 99% of the total reads. After quality control splicing, sequencing data were clustered into operational taxonomic units (OTUs) at 97% similarity. The obtained OTUs were annotated with the Silva database, and the alpha and beta diversity analyses were performed using R software version 4.2.1 and related packages [22].

## 3. Results and Discussion

### 3.1. Effects of Hg^2+^ on Microbial S^0^ Reduction and Hg^2+^ Fixation

**Figure 1:**
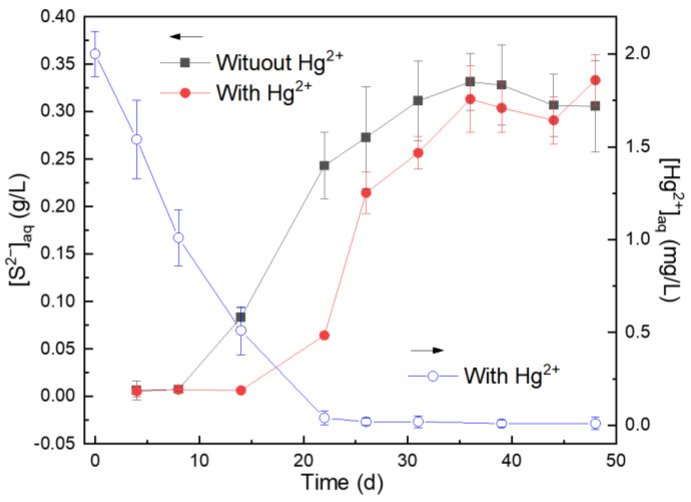
Changes in [S^2−^]_aq_ and [Hg^2+^]_aq_ in the solution during microbially anaerobic S^0^ reduction for the groups without and with Hg^2+^.

The [S^2−^]_aq_ for the experimental group without Hg^2+^ was significantly higher than that with the addition of Hg^2+^ at the early cultivation stage, and then gradually increased to the stationary stage with no significant difference for both groups in the later cultivation stage (Figure 1), indicating the microbial S^0^ reduction was inhibited initially by Hg^2+^, and the inhibition effect of Hg^2+^ on microbial cells was gradually relieved as the culture time increased. The [Hg^2+^]_aq_ for the group with Hg^2+^ showed a downward trend at days 0–22, and then decreased to approximately zero (Figure 1). These results indicated that the presence of Hg^2+^ can inhibit microbial S^0^ reduction at the early cultivation stages, resulting in the extension of the adaptation period for bacterial growth (Figure S2). Bacteria enter exponential growth after adapting to or gradually relieving Hg stress through bioremediation by mercury-resistant bacteria (Figure S2) [10]. Furthermore, Hg^2+^ in the solution can react with S^2−^ to produce HgS and can also be reduced to Hg^0^ by mercury-reducing bacteria [12,13,23], resulting in a decrease in [Hg^2+^]_aq_ in the solution and reducing the inhibition of Hg^2+^ on microbial cells.

### 3.2. Microbial Cell Morphology and Surface Composition

**Figure 2:**
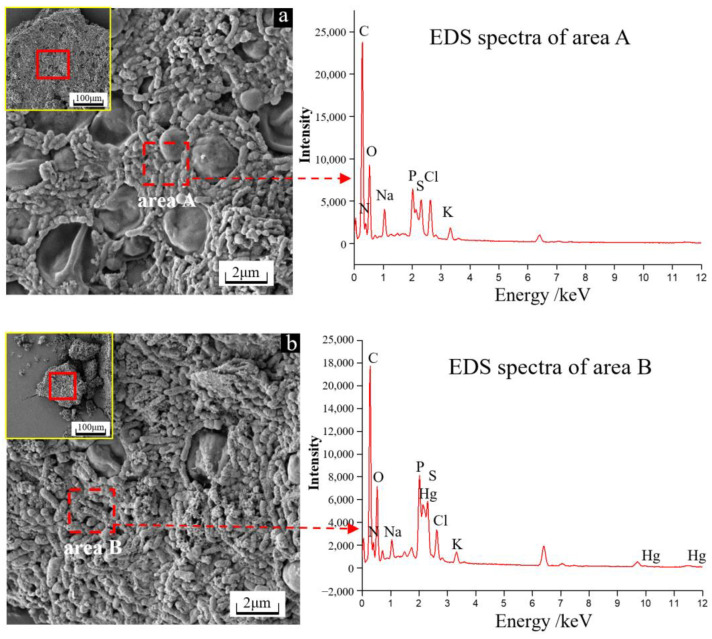
SEM images and EDS spectra of the microbial cells on day 48 for the groups without (**a**) and with (**b**) Hg^2+^.

According to the results in Figure 2a,b, the microbial cells were mainly rod shaped, spherical, and ellipsoidal. In the absence of Hg^2+^, the cell surface was smooth (Figure 2a), while in the presence of Hg^2+^, granular and flocculent substances were common around the cells (Figure 2b). The EDS analysis results showed relatively high proportions of C, N, O, P and S on the cells for each group, with 1.52% and 1.71% of S for the groups without and with Hg^2+^, respectively. In addition, in the presence of Hg^2+^, a small amount of Hg was also detected on the cell surface. These results indicated that for the group with the addition of Hg^2+^, microorganisms can enrich Hg through their cell surface substances and produce flocculent granules. Of note, the presence of Hg was closely related to S, which may be because the cell surface is rich in thiol groups (-SH), and the thiol groups can capture Hg^2+^ on the cell surface by forming S-Hg bonds with Hg^2+^ [24].

**Figure 3:**
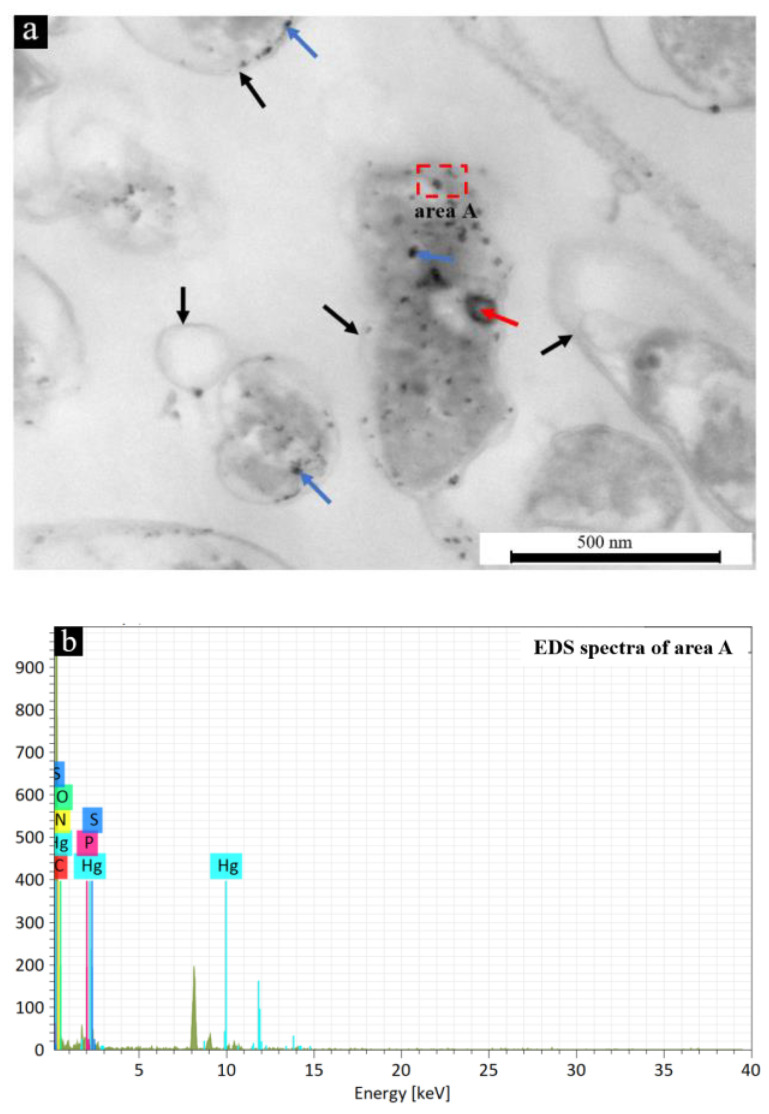
TEM image (**a**) and EDS spectra (**b**) of the cells on day 48 for the group with Hg^2+^, where the black, red and blue arrows in panel (**a**) show the cell wall, the genetic material and the formed Hg-containing particles, respectively.

To illustrate the utilization of Hg by microbial cells, the internal structure and elemental distribution of cells for the group with Hg^2+^ were analyzed by TEM-EDS (Figure 3). Figure 3a shows that the outline of the cell section was clearly visible, and many small black depositional dots accumulated on the cell wall and inside the cell. EDS of the arbitrarily selected area (dotted rectangle in Figure 3a) shows that the black depositional dots in the bacterial cell contained a large amount of Hg and S (Figure 3b), which further proved the process by which bacteria capture Hg^2+^ through extracellular polymer substances (EPS) and transport it to the interior of the cell for utilization.

**Figure 4:**
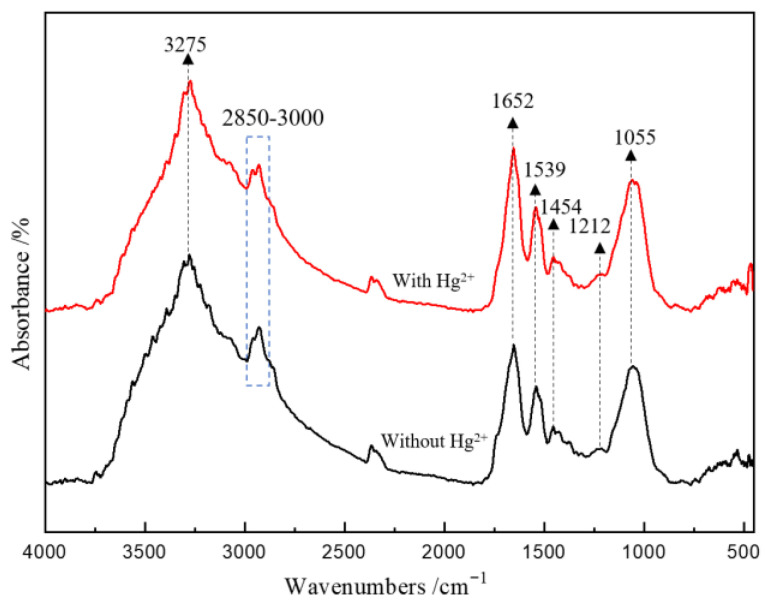
The FT-IR spectra of AMD-enriched microbial cells at day 48 for the groups without and with Hg^2+^.

The FT-IR spectra of the cells for the groups without and with Hg^2+^ are shown in Figure 4. The cells for all groups had absorption bands with different intensities at 3275, 2850–3000, 1652, 1539, 1454, 1212 and 1055 cm^−1^. Among them, the band at 3275 cm^−1^ is due to the stretching vibration of the O-H bond, the bands at 2850–3000 cm^−1^ are due to the vibration of -CH_2_ and -CH_3_ in fatty acids, the bands at 1652 cm^−1^ and 1539 cm^−1^ are associated with C=O and N-H in protein amide bonds, the band at 1454 cm^−1^ is due to the vibration of the benzene ring skeleton, the band at 1212 cm^−1^ is assigned to the stretching vibration of the C-O group, and the band at 1055 cm^−1^ is associated with S=O [25]. Compared with the FT-IR results of microbial cells without Hg^2+^, it was found that for the group with Hg^2+^, the intensity of the bands at 2850–3000 cm^−1^ and 1212 cm^−1^ apparently decreased, indicating that the existence of Hg^2+^ resulted in the decreased expression of some polysaccharides and lipids in bacteria. In addition, the bands at 1652 and 1539 cm^−1^ became sharper and increased in intensity for the group with Hg^2+^, indicating an increase in cell surface protein expression. These results are very likely to be related to the Hg tolerance mechanism of the AMD enrichment culture; that is, the microorganism secretes more -SH-containing extracellular proteins under the stress of Hg^2+^ and adsorbs and immobilizes Hg^2+^ to attenuate or mitigate its toxicity [26,27].

### 3.3. Surface Morphology and Composition of Solid Particulate 

**Figure 5:**
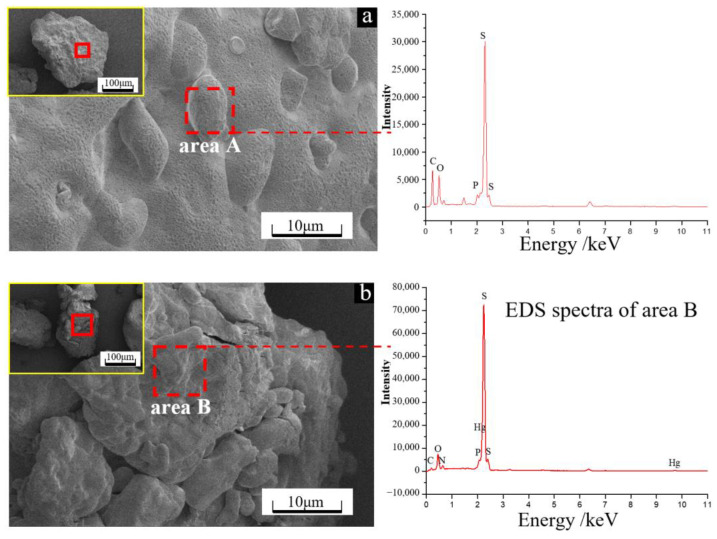
SEM image and EDS spectra of the solid particulate on day 48 for the groups without (**a**) and with (**b**) Hg^2+^.

In the absence of Hg^2+^, the surface morphology of the solid particulate was relatively intact with concave traces of depressions eroded by bacteria (Figure 5a), while in the presence of Hg^2+^, it was relatively rough with loose particles (Figure 5b). The EDS results showed that in the absence of Hg^2+^, the surface of the solid particulate mainly consisted of S (49.7%), C (70.8%) and O (14.0%), while in the presence of Hg^2+^, the surface contained small amounts of Hg in addition to S, C and O, and the proportions of S, C and O decreased. These results indicated that in the absence of Hg^2+^, microbial cells might adhere and erode to the sulfur surface, forming corrosion pits on the surface and increasing extracellular polymers, while in the presence of Hg^2+^, microbial cells might enter the sulfur particles for action, resulting in loose granules on the surface.

**Figure 6:**
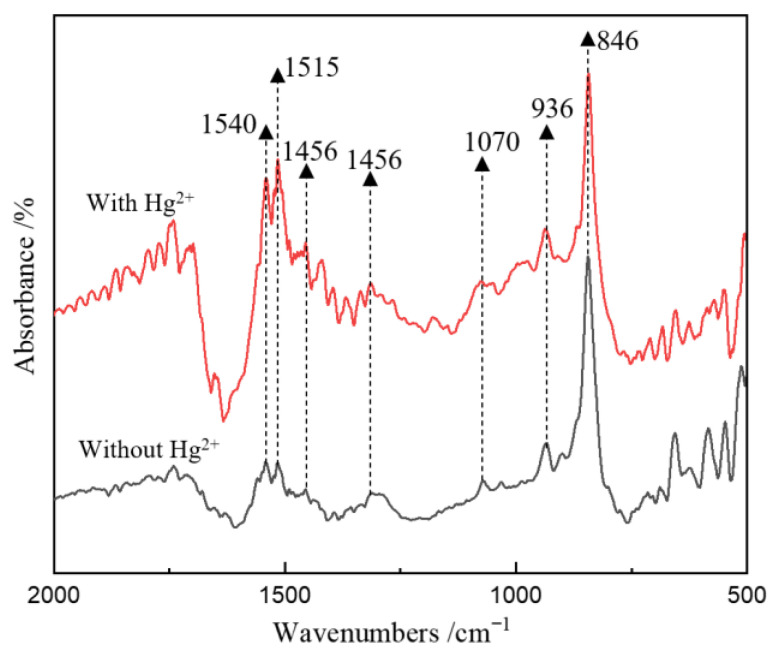
FT-IR spectra of the solid particulate at day 48 for the groups without and with Hg^2+^.

The surface composition of the particulate matter during the experiment was further analyzed by FT-IR spectroscopy, and the results are shown in Figure 6. According to a previous study [28], the FT-IR band at 846 cm^−1^ is the characteristic peak of S^0^ (Figure 6). For both groups without and with Hg^2+^, vibration bands related to phosphate groups and hydrocarbons (at 900 ~ 1500 cm^−1^) and protein amide bonds (at 1515 and 1540 cm^−1^) appeared (Figure 6), indicating the adsorption of microbes on the S^0^ surface. Notably, for the group with Hg^2+^, the intensity of the bands at 900~1500 cm^−1^ and the bands at 1515 and 1540 cm^−1^ are higher than that for the group without Hg^2+^, indicating more microbial cells adsorbed on the S^0^ surface.

### 3.4. Dynamics of C, S and Hg Speciation on Solid Particulate Surface

**Figure 7:**
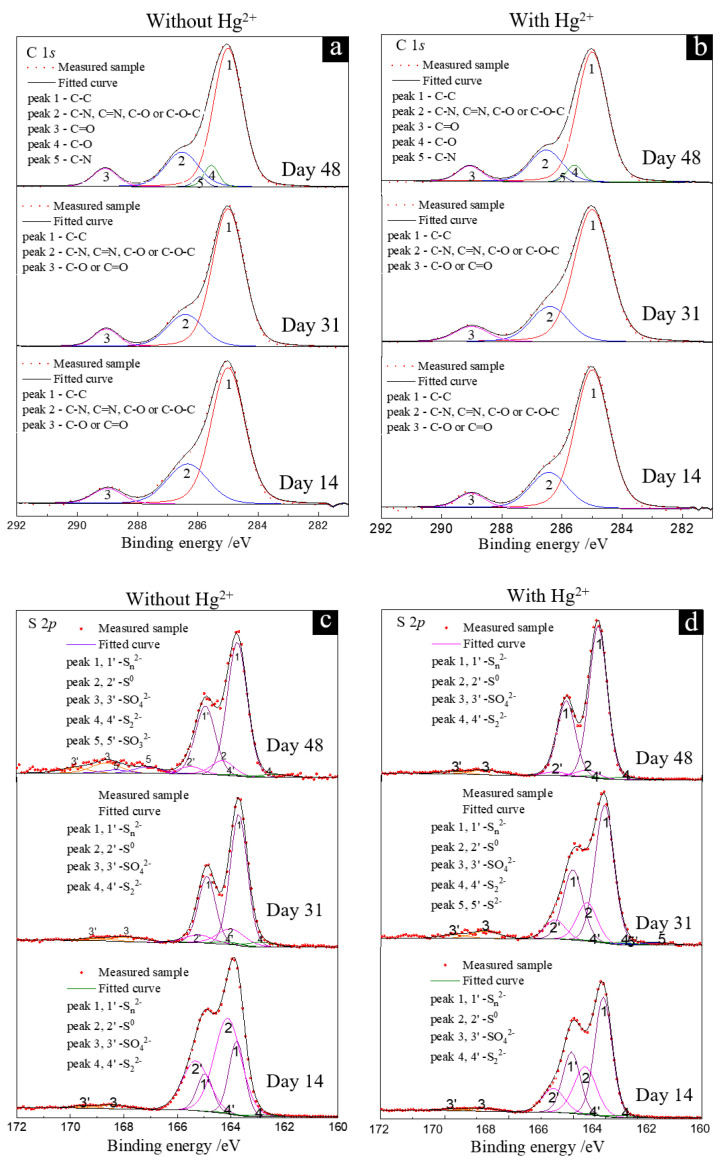

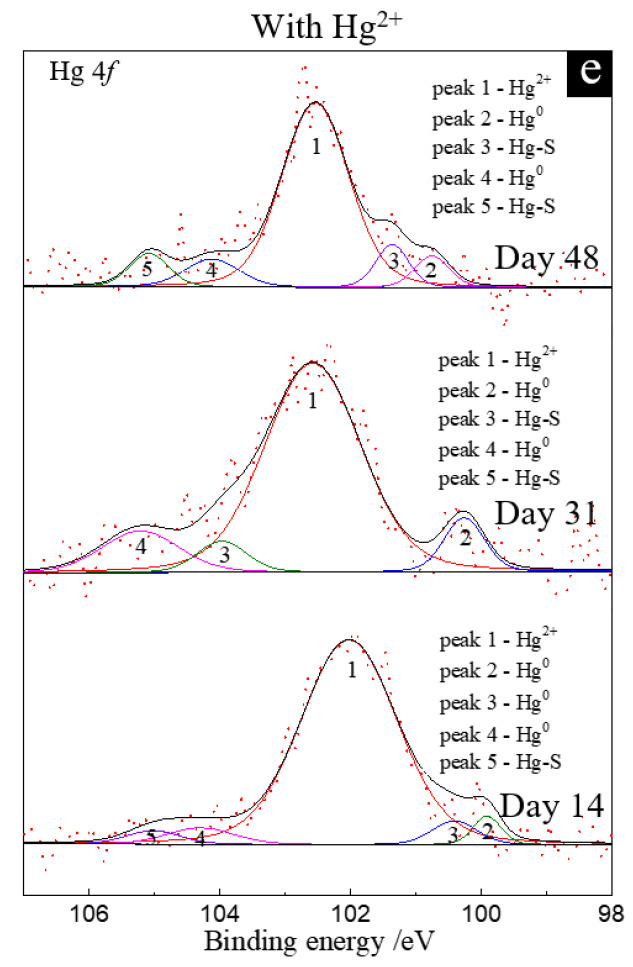
The C 1*s* (**a**,**b**), S 2*p* (**c**,**d**) and Hg 4*f* (**e**) XPS spectra of the solid particulate on days 14 (**a**), 31 (**b**) and 48 (**c**) for the groups without (**a**,**c**) and with (**b**,**d**,**e**) Hg^2+^.

During the S^0^ reduction by the AMD enrichment culture, complex speciation transformations of C, S and Hg occurred, which were closely related to the changes in solution physical and chemical properties, the mineral and microbial surface structure, and the microbial community composition. To analyze the fate of Hg and the formation process and mechanism of secondary products during the anaerobic reduction of S^0^ by the AMD enrichment culture, the C/S/Hg speciation transformation on the solid particulate surface was analyzed based on XPS spectroscopy and the results are shown in Figure 7. 

The fitting results of the C 1*s* XPS spectra (Figure 7a,b) showed that the main carbon species on the surface of the solid particulate included C-C/C-H (284.8 eV), C-O (286.4 eV), and O-C=O (289.0 eV) [29,30]. By comparing the C 1*s* XPS spectra for both groups on days 14, 31 and 48, it was found that with increasing culture time, the intensity of fitting peak 3 of C = O increased, and new peaks 4 (C=O) and 5 (C-N) appeared at 285.5 eV and 285.9 eV, indicating the existence of microbial substances on the surface of solid particulate, which are consistent with the results of the FT-IR spectra. Notably, the intensities of fitting peaks 2 and 3 at day 14 for the group in the presence of Hg^2+^ were lower than those without Hg^2+^, while their intensities were similar for both groups at days 31 and 48, indicating that the growth of the AMD enrichment culture gradually returned to normal after adaptation to Hg^2+^ and produced more EPS on the surface of the solid particulate. 

The fitting results of the S 2*p* XPS spectra (Figure 7c,d) showed that the S species on the S^0^ surface were mainly composed of S_n_^2−^ (163.6 ± 0.3 eV), S^0^ (164.2 ± 0.4 eV), SO_4_^2−^ (168.0 ± 0.3 eV), S_2_^2−^ (162.6 ± 0.3 eV), S^2−^ (161.5 ± 0.3 eV) and SO_3_^2−^ (166.8 ± 0.4 eV) [31]. By comparing the S 2*p* XPS spectra on days 14, 31 and 48, it was found that the characteristic peak of S^0^ gradually decreased during the utilization of S^0^ for both groups, accompanied by a gradual increase in the intensity and proportion of the peak at 163.8 eV, indicating that S^0^ is continuously reduced to S_n_^2−^, S_2_^2−^ and S^2−^. Notably, by comparing the group without Hg^2+^, the intensity of the S_n_^2−^ peak for the group with Hg^2+^ was significantly lower than that of the S^0^ peak at day 14, which was probably due to the fixation of Hg^2+^ on the solid S^0^ surface.

The fitting results of the Hg 4*f* XPS spectra show that the Hg species on the surface of the solid particulate mainly exist in the form of Hg^2+^, Hg^0^ and Hg-S (Figure 7e). By comparing the Hg 4*f* XPS spectra on days 14, 31 and 48, it was found that with increasing culture time, new peaks appeared at approximately 104.5 eV (Hg-S) and 100.0 eV (Hg^0^) for the group with Hg^2+^, and the peak position of Hg^2+^ (peak 1) moved to the right, indicating a trend of Hg^2+^ reduction. The formation of Hg^0^ by microbial Hg^2+^ reduction was also observed by Raman spectroscopy (Figure S3), which is considered to be an important detoxification pathway for microbial growth in the presence of Hg^2+^ [11,12,13]. 

Based on the C 1*s*, S 2*p* and Hg 4*f* on the surface of the solid particulate, it can be inferred that the speciation transformation of C, S and Hg is closely related to the anaerobic S^0^ reduction process by the AMD enrichment culture. Under anaerobic conditions, the interaction between the AMD enrichment culture and S^0^ leads to the transformation of S and Hg species, the reduction of S^0^ in the solid phase and an increase in [S^2−^]_aq_ in the solution. In addition, combined with EDS analysis of AMD-enriched cells and solid particulate, it can be confirmed that the fixation of Hg^2+^ further affects the fate behavior of Hg, making it transfer from solution to substrate sulfur and the cell surface. HgS and Hg^2+^ can further combine with EPS and are transported to cells and transformed into Hg^0^ through microbial action [32].

### 3.5. Microbial Community Structure 

To further illustrate the correlation between anaerobic S^0^ reducing microorganisms and Hg^2+^ transformation, the microbial community structures for the groups without and with Hg^2+^ were analyzed based on 16S rDNA high-throughput sequencing, and the number of OTUs was 501 and 490, respectively. The richness and Simpson indexes of the OTUs were used for the alpha-diversity analysis, and principal component analysis (PCA) was used for the beta-diversity analysis (Figure S4). The richness index can characterize the richness of the microbial community, and the Simpson index can reflect the evenness of the microbial community. The results in Figure S4a,b show that the richness index for both groups was not significantly different, while the Simpson index for the group with Hg^2+^ was significantly lower than that for the group without Hg^2+^, and the results in Figure S4c show that the difference of the microbial community for the group with Hg^2+^ is relatively small by compassion with that without Hg^2+^. These results indicated that the addition of Hg^2+^ did not change the richness of the microbial community but decreased the evenness of microbial diversity, and had enrichment effect on microbial community. 

**Figure 8:**
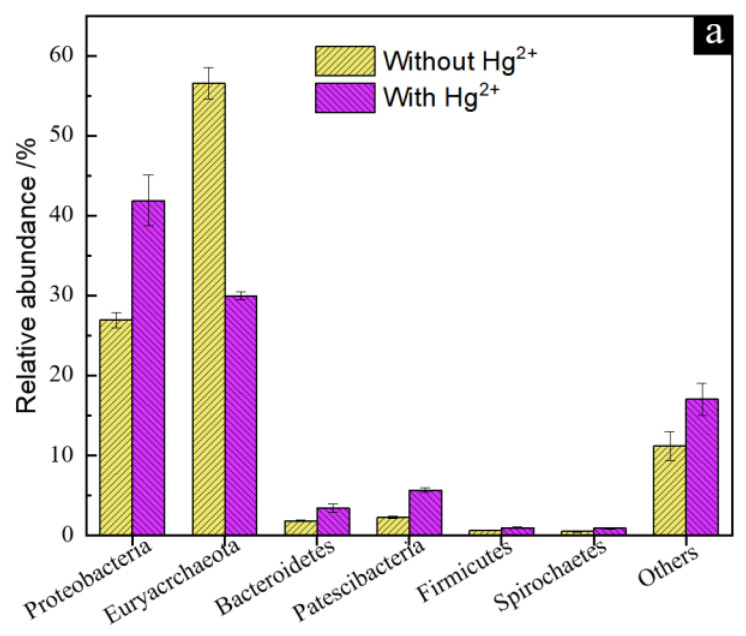

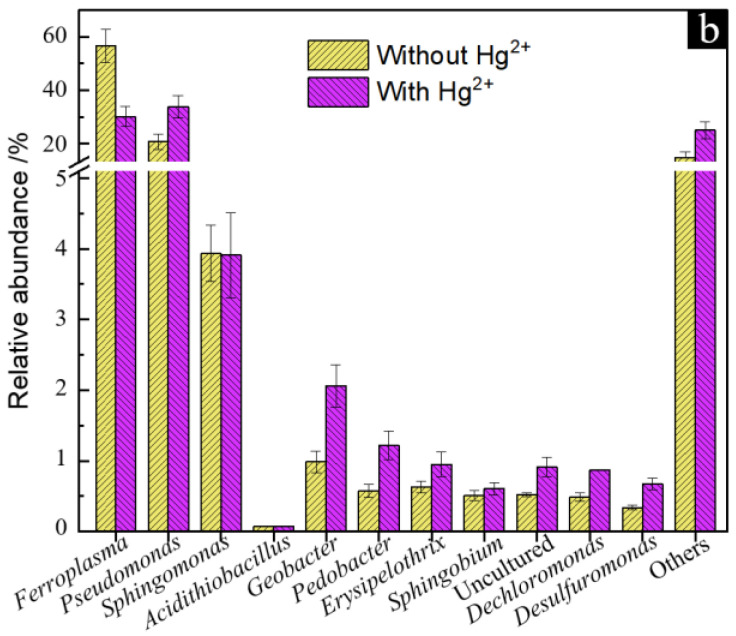
The microbial community composition histogram at the phylum level (**a**) and the genus level (**b**) for the groups without and with Hg^2+^.

The microbial OTUs can be assigned to 29 phyla, 62 classes, 135 orders, 149 families and 170 genera for the group without Hg^2+^, and 35 phyla, 63 classes, 131 orders, 152 families and 174 genera for the group with Hg^2+^. The relative abundance results of the microbial community at the phylum level show that Proteobacteria and Euryarchaeota were dominant for both cases without and with Hg^2+^ (Figure 8a). By comparing with the group without Hg^2+^, the relative abundance of Proteobacteria, Patescibacteria and Bacteroides for the group with Hg^2+^ was significantly increased, and the relative abundance of Euryarchaeota decreased, indicating that Euryarchaeota is significantly inhibited by Hg^2+^, while Proteobacteria, Patescibacteria and Bacteroides can tolerate Hg^2+^ for growth and metabolism. Liu et al. [33] and Vishnivetskaya et al. [34] found dominant mercury-resistant bacteria such as Proteobacteria and Euryarchaeota in Hg-contaminated paddy soil. Patescibacteria widely exists in anaerobic environments such as groundwater and sediments and has very small cells and genomes, simple structures, and metabolic functions, which may explain why it can tolerate Hg^2+^ and become the dominant strain [17]. In addition, Bacteroidetes, Firmicutes and Spirochaetes have been reported to be resistant to Hg^2+^ or able to survive in extreme environments rich in heavy metal ions [35,36,37,38].

The microorganisms with a relative abundance of more than 0.1% at the genus level were further selected to show their differences (Figure 8b). Compared with the group without Hg^2+^, the relative abundance of *Pseudomonas*, *Geobacter*, *Pedobacter*, *Dechloromonas* and *Desulfuromonas* for the group with Hg^2+^ increased, and the relative abundance of *Ferroplasma* decreased. Previous studies have shown that *Geobacter* and *Desulfuromonas* have the ability of mercury methylation [39], and *Pseudomonas* has the ability of methylmercury demethylation and mercury reduction through its *mer* operon [40,41]. Of note, although bacteria with mercury methylation ability are abundant, the MeHg species was not detected in the solution of the culture medium for the group with Hg^2+^ added, which was probably because almost all the dissolved Hg^2+^ was transformed to HgS and Hg^0^, resulting in the relatively low concentration of MeHg. In addition, the model bacterium *Geobacter sulfurereducens* is an iron-reducing bacterium with a sulfur reduction function that can reduce Hg^2+^, and *Pseudomonas* and *Desulfuromonas* are key sulfur-reducing bacteria that can reduce S^0^ to H_2_S under anaerobic conditions [42,43]. These results indicated that the existence of Hg^2+^ could significantly change the community structure of the AMD enrichment culture under anaerobic S^0^ reduction, leading to microbial succession to the community with Hg^2+^ transformation function.

## 4. Environmental Significance

The above results show that the transformation of Hg^2+^ in the process of anaerobic S^0^ reduction by an AMD environmental enrichment culture is closely related to the microbial sulfur reduction function, and the proposed correlation mechanism is shown in Figure S5. It was concluded that the anaerobic S^0^ reduction process of the AMD environmental enrichment culture was inhibited in the presence of Hg^2+^. However, with the development of culture, the AMD enrichment culture could transform Hg^2+^ into Hg^0^ and HgS, gradually relieving the inhibition of Hg^2+^ in the environment, and microbial S^0^ reduction also recovered rapidly. The process of disinhibition is closely related to the extracellular substances of the enrichment culture, especially the -SH groups of proteins. Of note, the genera *Pseudomonas*, *Geobacter*, *Pedobacter*, *Dechloromonas* and *Desulfuromonas*, with mercury reduction and sulfur reduction functions, occupied the main community for the cases with the addition of Hg^2+^, resulting in the enhancement of Hg resistance and the S^0^ reduction function of the microbial community. Anaerobic S^0^ reduction microorganisms continuously reduced S^0^ to S^2−^, and the produced S^2−^ could combine with Hg^2+^ in the solution and continuously generate HgS on the solid particulate and cell surface, which promoted Hg^2+^ transformation. These processes may be the key driving force for the transformation and fixation of soluble Hg^2+^ in the anaerobic S^0^ reduction process by AMD enrichment culture. Hg-reducing microorganisms can also promote Hg^2+^ transformation by reducing Hg^2+^ to Hg^0^, and the evenness of the microbial community related to Hg reduction will decrease. The conversion of the AMD enrichment culture to a microbial community structure with Hg^2+^ transformation functions could improve the adaptability of the AMD environmental enrichment culture to Hg^2+^, which is an important detoxification mechanism.

## 5. Conclusions

The microbial growth and S^0^ reduction of the AMD enrichment culture were significantly inhibited in the early stage of cultivation by the addition of Hg^2+^. With increasing culture time, microorganisms gradually tolerate and release the inhibitory effect of Hg^2+^ in the environment. On the one hand, microorganisms can enrich Hg^2+^ through their cell surface substances and produce flocculent and granular particles. On the other hand, bacteria can capture Hg^2+^ through extracellular polymers and further transport it to the interior of cells. Through microbial mercury reduction, Hg^0^ can be formed in the cell. In the presence of Hg^2+^, the evenness of the microbial community decreased, and the anaerobic microorganisms related to mercury metabolism and sulfur reduction gradually became dominant. The dominant microorganisms with S^0^ reduction functions are closely related to mercury transformation and are the key driving force for the transformations of solid particulate and cells and the fixation of Hg^2+^.

## Data Availability

Sequence data were deposited at NCBI under accession number PRJNA910676.

## Supplementary Materials

The following supporting information can be downloaded at: https://www.mdpi.com/article/10.3390/microorganisms11010072/s1, Figure S1: Schematic diagram of the bioreactor used for microbial enrichment in AMD environment; Figure S2: Changes in the cell density, [S^2−^]_aq_ and total Hg during microbially anaerobic S^0^ reduction for the groups with 0, 1.0, 2.0 and 5.0 mg/L Hg^2+^; Figure S3: The Raman spectra of the solid particulate on days 14, 31 and 48 for the reference samples, and for the groups without and with Hg^2+^; Figure S4: The richness index richness and Simpson index diversity of the microbial community for the groups without and with Hg^2+^; Figure S5: The proposed correlation mechanism between Hg^2+^ transformation and anaerobic S^0^ reduction by AMD enrichment culture.
