# Supplementary material for: The Transformation of Hg2+ during Anaerobic S0 Reduction by an AMD Environmental Enrichment Culture
Source: Microorganisms. 2022 Dec 27;11(1):72. doi: 10.3390/microorganisms11010072 (PMC9865316; doi:10.3390/microorganisms11010072)
Supplement: Supplementary file 1 [file microorganisms-11-00072-s001.zip › microorganisms-2092314-supplementary.pdf]

Supplementary materials

for

The transformation of Hg^{2+} during anaerobic S^0 reduction by an AMD environmental enrichment

Yuhang Zhou[1†], Yue Liu[1†], Hongchang Liu[1,2*], Zhenyuan Nie[1,2], Yirong Wang[1], Lu Chen[1]

[1]. School of Minerals Processing and Bioengineering, Central South University, Changsha 410083, China

[2]. Key Lab of Biometallurgy of Ministry of Education of China, Central South University, Changsha 410083, China

[†] These authors contributed equally to this work

* Correspondence: Hongchang Liu, Email: hchliu2050@csu.edu.cn

The supplementary materials contain five supplementary figures.

Figure S1

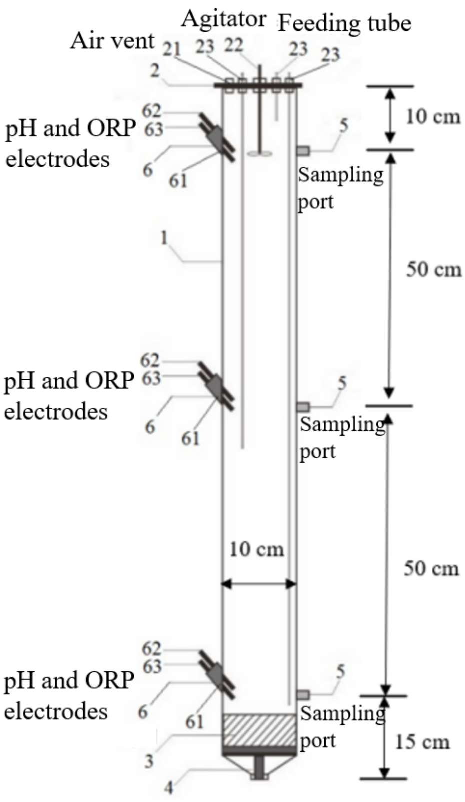

Figure S1 Schematic diagram of the bioreactor used for microbial enrichment in AMD environment.

Figure S2

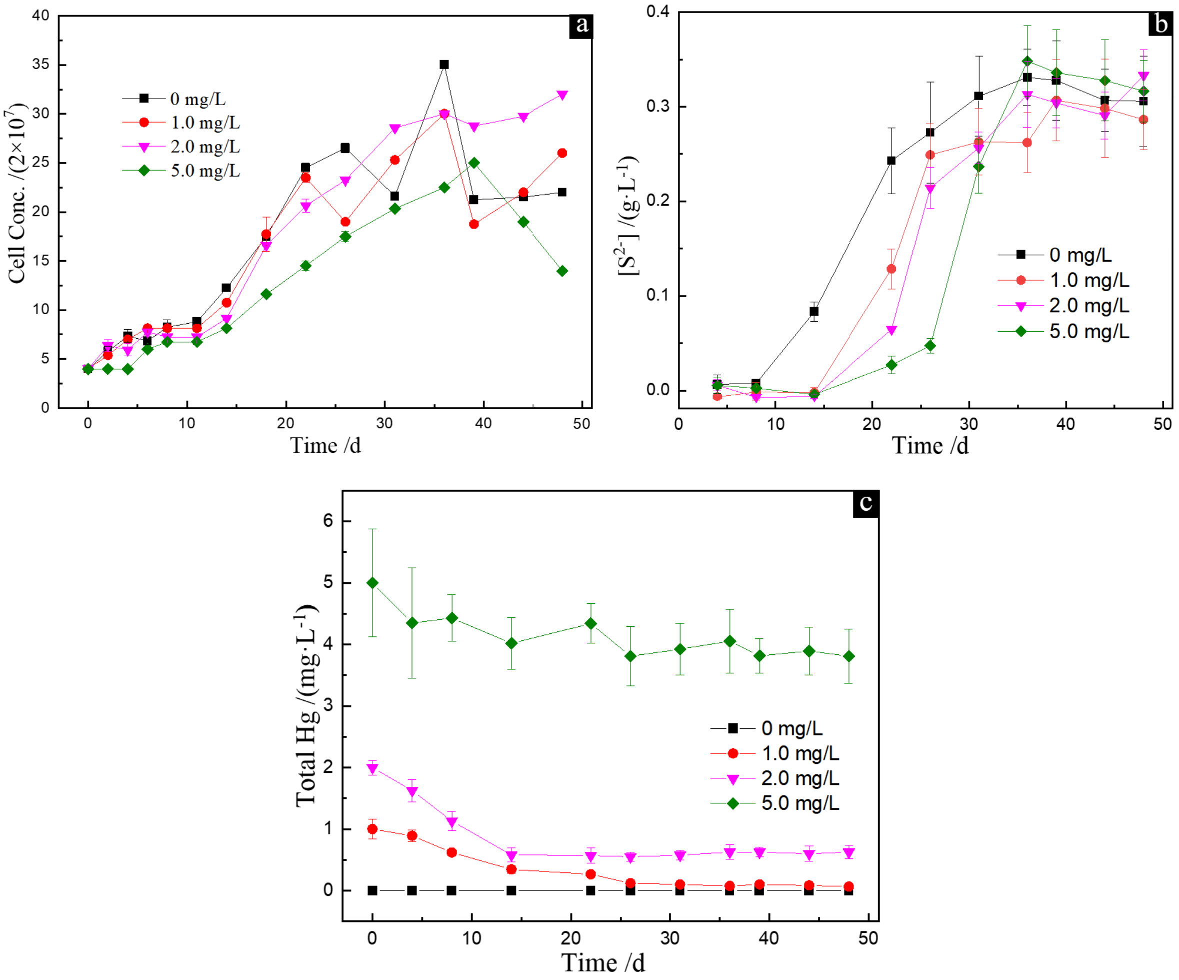

Figure S2. Changes in the cell density (a), $[S^{2-}]_{aq}$ (b) and total Hg (c) during microbially anaerobic S^0 reduction for the groups with 0, 1.0, 2.0 and 5.0 mg/L Hg^{2+}

Note. The cell concentration for all groups gradually increased and then fluctuated (Figure S2a). For the groups with the addition of 0, 1.0, and 2.0 mg/L Hg^{2+}, there were no significant differences in cell concentrations on days 0 to 18, while for the 5.0 mg/L Hg^{2+} group, the cell concentration was significantly lower than that of the other groups, indicating that the growth of microorganisms was significantly inhibited. Of note, in the presence of 2.0 mg/L Hg^{2+}, the cell concentration was significantly higher than that of other groups after 40 days, indicating that the microbial growth was better than that of the other groups.

Figure S2b shows that the $[S^{2-}]_{aq}$ for the experimental group without Hg^{2+} was significantly higher than those with the addition of Hg^{2+} at the early cultivation stage, and then gradually increased to the stationary stage with no significant difference for different groups in

the later cultivation stage, indicating the microbial S^0 reduction was inhibited by Hg^{2+}, and the inhibition effect of Hg^{2+} on microbial cells was gradually relieved as the culture time increased.

Figure S2c shows the total Hg in the solution after filtering to remove S^0 substrate sediment. The total Hg concentrations for all groups with the addition of Hg^{2+} decreased, and it decreased to 0.07, 0.63 and 3.42 mg/L for the groups with 1.0, 2.0 and 5.0 mg/L Hg^{2+}, respectively. Notably, the total Hg concentrations for the groups with Hg^{2+} is not zero, especially for the groups with the addition of 2.0 and 5.0 mg/L Hg^{2+}. However, the dissolved Hg^{2+} in the solution (after removing cells and micro particles through high-speed centrifugation) was basically zero for all groups at the late stage of cultivation, indicating these Hg^{2+} adsorbed onto the cells or forming micro particles containing mercury onto the cells.

Figure S3

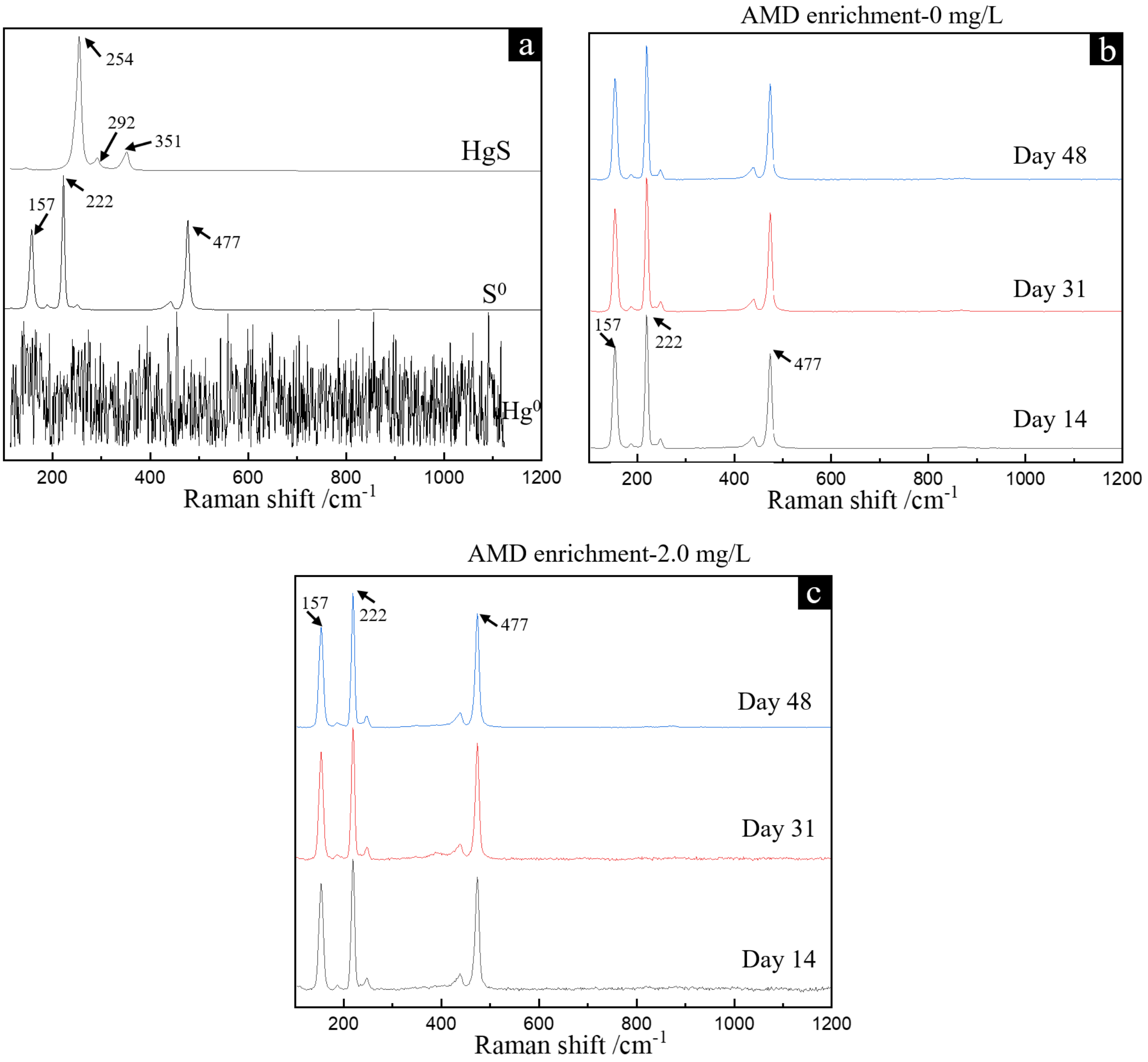

Figure S3. The Raman spectra of the solid particulate on days 14, 31 and 48 for the reference samples (a), and for the groups without (b) and with (c) Hg^{2+}.

Note. Figure S3a shows the Raman characteristic peaks for the three reference samples (HgS, S^0 and Hg^0), in which the characteristic peaks of HgS are 254, 292 and 351 cm^{-1}, the characteristic peaks of S^0 are 157, 222 and 477 cm^{-1}, and the Raman spectrum of Hg^0 shows more miscellaneous peaks. The results in Figure S3b-c show that for all groups without and with Hg^{2+}, obvious S^0-related Raman peaks occurred at 157, 222 and 477 cm^{-1}. By comparison with the Raman spectra on days 14, 31 and 48 in the presence of Hg^{2+}, it was found that the signal of the miscellaneous peaks decreased with increasing incubation time, indicating that Hg^0 on the surface of the solid particulate gradually decreased with increasing incubation time.

Figure S4

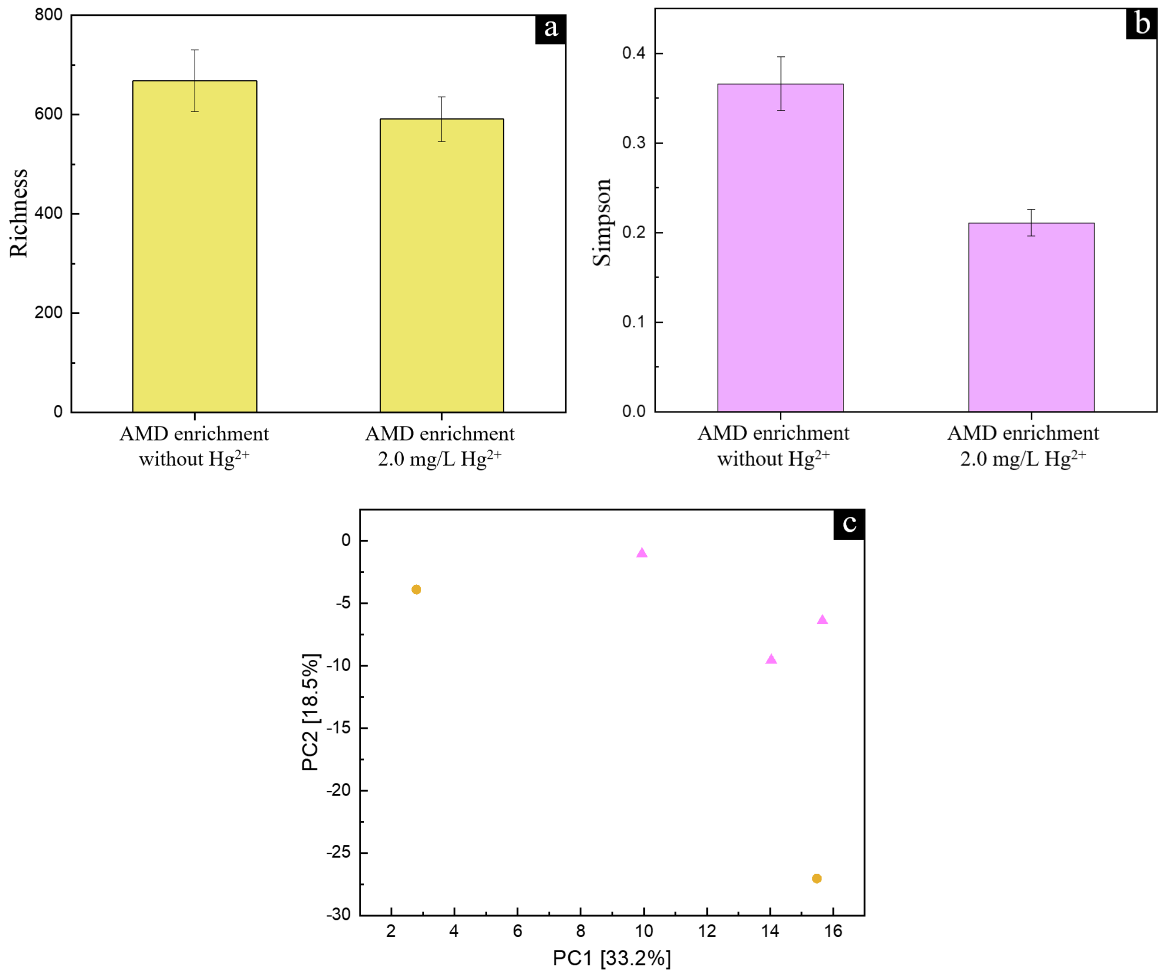

Figure S4. The Richness index richness (a), Simpson index diversity (b) and PCA (c) of the microbial community for the groups without and with Hg^{2+}.

Figure S5

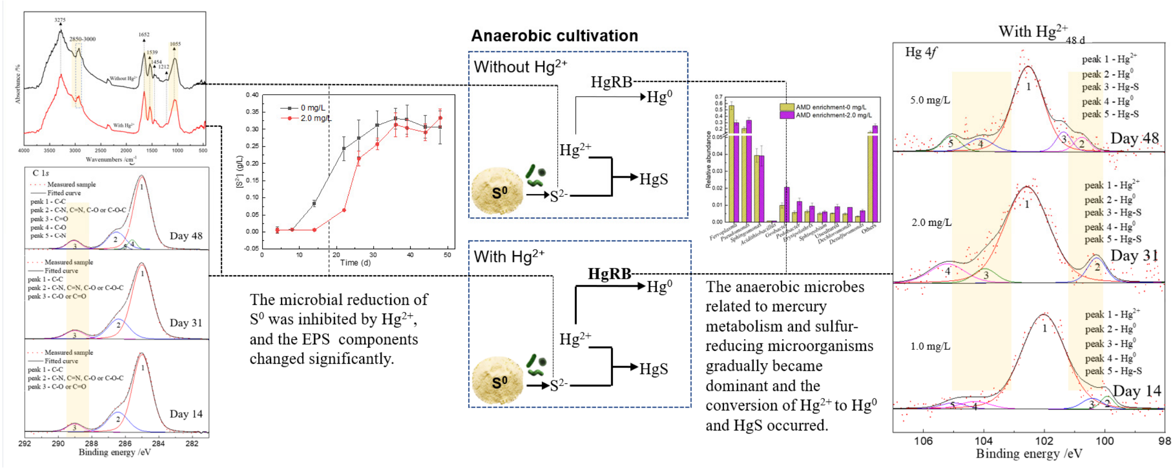

Figure S5. The proposed correlation mechanism between Hg^{2+} transformation and anaerobic S^0 reduction by an AMD enrichment culture. The thick lines in the dashed box indicate that the formation of the intermediate (S^{2-}) and/or products (Hg^0 and HgS) was enhanced.
